# Two Phosphoglucomutase Paralogs Facilitate Ionophore-Triggered Secretion of the *Toxoplasma* Micronemes

**DOI:** 10.1128/mSphere.00521-17

**Published:** 2017-11-29

**Authors:** Sudeshna Saha, Bradley I. Coleman, Rashmi Dubey, Ira J. Blader, Marc-Jan Gubbels

**Affiliations:** a Department of Biology, Boston College, Chestnut Hill, Massachusetts, USA; b Department of Microbiology and Immunology, University at Buffalo School of Medicine, Buffalo, New York, USA; Carnegie Mellon University

**Keywords:** PRP1, *Toxoplasma gondii*, calcium, micronemes, parafusin, phosphoglucomutase

## Abstract

Paralogs of the widely prevalent phosphoglucomutase (PGM) protein called parafusin function in calcium (Ca^2+^)-mediated exocytosis across eukaryotes. In *Toxoplasma gondii*, the parafusin-related protein 1 (PRP1) has been associated with Ca^2+^-dependent microneme organelle secretion required for essential processes like host cell invasion and egress. Using reverse genetics, we observed PRP1 to be dispensable for completion of the lytic cycle, including host cell invasion and egress by the parasite. However, the absence of the gene affected increased microneme release triggered by A23187, a Ca^2+^ ionophore used to raise the cytoplasmic Ca^2+^ concentration mimicking the physiological role of Ca^2+^ during invasion and egress. The basal levels of constitutive microneme release in extracellular parasites and phosphatidic acid-triggered microneme secretion were unaffected in the mutant. The phenotype of the deletion mutant of the second PGM-encoding gene in *Toxoplasma*, PGM2, was similar to the phenotype of the PRP1 deletion mutant. Furthermore, the ability of the tachyzoites to induce acute infection in the mice remained normal in the absence of both PGM paralogs. Our data thus reveal that the microneme secretion upon high Ca^2+^ flux is facilitated by the *Toxoplasma* PGM paralogs, PRP1 and PGM2. However, this protein-mediated release is neither essential for lytic cycle completion nor for acute virulence of the parasite.

**IMPORTANCE** Ca^2+^-dependent exocytosis is essential for the life cycle of apicomplexan parasites. *Toxoplasma gondii* harbors a phosphoglucomutase (PGM) ortholog, PRP1, previously associated with Ca^2+^-dependent microneme secretion. Here it is shown that genetic deletion of either PRP1, its PGM2 ortholog, or both genes is dispensable for the parasite’s lytic cycle, including host cell egress and invasion. Depletion of the proteins abrogated high Ca^2+^-mediated microneme secretion induced by the ionophore A23187; however, the constitutive and phosphatidic acid-mediated release remained unaffected. Secretion mediated by the former pathway is not essential for tachyzoite survival or acute *in vivo* infection in the mice.

## Abstract (precis)

Ca^2+^-dependent exocytosis is essential for the life cycle of apicomplexan parasites. *Toxoplasma gondii* harbors a phosphoglucomutase (PGM) ortholog, PRP1, previously associated with Ca^2+^-dependent microneme secretion. Here it is shown that genetic deletion of either PRP1, its PGM2 ortholog, or both genes is dispensable for the parasite’s lytic cycle, including host cell egress and invasion. Depletion of the proteins abrogated high Ca^2+^-mediated microneme secretion induced by the ionophore A23187; however, the constitutive and phosphatidic acid-mediated release remained unaffected. Secretion mediated by the former pathway is not essential for tachyzoite survival or acute *in vivo* infection in the mice.

## INTRODUCTION

The apicomplexan parasite *Toxoplasma gondii* has infected one in every three humans globally with most acute infections being mild or asymptomatic (1). However, severe disease occurs congenitally in pregnant women (2) and in immunocompromised patients (e.g., AIDS patients) where reactivation of a chronic infection can result in life-threatening encephalitis or myocarditis (3). The clinical manifestation is the consequence of tissue destruction resulting from the repetitive cycles of active invasion, intracellular replication, and egress that make up the lytic cycle of the parasite. As a result, host cell invasion is a critical event in the pathogenesis of this obligate parasite (4). Unlike some other intracellular pathogens, the invasion by *Toxoplasma* is a parasite-directed event requiring its motility and sequential release of three secretory organelles (5, 6). The secretion of the first organelle, the micronemes, is regulated by intracellular calcium (Ca^2+^) fluxes (7–9); however, the molecular mechanism underlying this regulation, especially the final events facilitating exocytosis or egress, is still incompletely understood.

The phosphoglucomutase (PGM) family comprises enzymes that interconvert glucose-1-phosphate and glucose-6-phosphate, thereby linking the cytosolic processes of glycogenolysis and glycolysis. However, a PGM ortholog known as parafusin, or PFUS, is present in many eukaryotes, including ciliates, yeast *Saccharomyces cerevisiae*, and humans, and functions in Ca^2+^-mediated signaling (10–14). The *Toxoplasma* genome encodes two PGM paralogs: PGM1 (accession no. TGME49_285980), also referred to as parafusin-related protein 1 (PRP1), and PGM2 (TGME49_318580) (15). In the ciliate *Paramecium*, PFUS has been associated with Ca^2+^-mediated exocytosis of dense core secretory vesicles (DCSVs) where it forms a scaffold on the vesicles and is involved in membrane fusion (16, 17, 18). Furthermore, PFUS has a role in DCSV assembly in both *Paramecium* (19) and *Tetrahymena* (20). Mechanistically, PFUS is hypothesized to impact the localized release of the matured DCSVs (17) through transient Ca^2+^-dependent phosphorylation during DCSV exocytosis; in its phosphorylated state, PFUS associates with the vesicles closest to the plasma membrane, priming them for secretion. For actual membrane fusion to occur, PFUS has to be dephosphorylated through the protein phosphatase calcineurin (17).

DCSV release in ciliates has been related to Ca^2+^-mediated microneme secretion in apicomplexan parasites (21). Germane to *Toxoplasma*, the ortholog PRP1 has been shown to localize to the most apical micronemes, and its phosphorylation status has been suggested to be Ca^2+^ dependent. Furthermore, through heterologous evaluation of PRP1 in *Paramecium*, an orthologous function in Ca^2+^-mediated microneme secretion has been proposed (22, 23). However, we recently reported that microneme secretion itself is independent of calcineurin (24), thereby suggesting an incomplete mechanistic orthology between ciliate DCSV release and microneme secretion in *Toxoplasma*.

Here we directly evaluated the roles of PRP1 and PGM2 through gene deletions in *Toxoplasma*. We show that both paralogs are required for microneme secretion in response to a high Ca^2+^ concentration stimulated by an ionophore, which mimics the cellular status during host cell invasion and egress. However, both paralogs are dispensable for constitutive microneme secretion accompanying gliding motility between host cells. Strikingly, tachyzoites devoid of the paralogs are completely viable *in vitro* and during acute mouse infections. These data suggest that the *Toxoplasma* PGMs are dispensable for Ca^2+^-dependent exocytosis and for the successful completion of the lytic cycle.

## RESULTS

### PRP1 is conserved across the coccidia.

**FIG 1:**
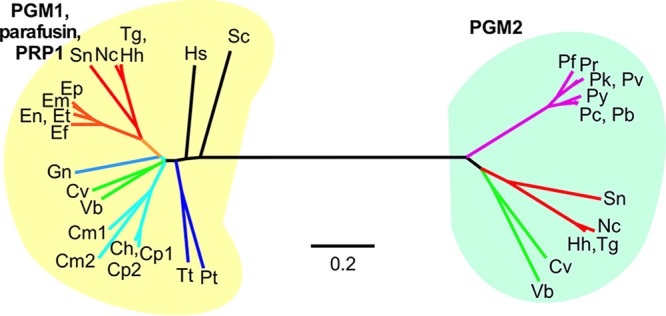
Phylogeny of PGMs in the Apicomplexa and their relatives. Unrooted tree based on alignment of PGM1 and PGM2 sequences from the following organisms: *Toxoplasma gondii* (Tg), *Neospora canimun* (Nc), *Hammondia hammondi* (Hh), *Sarcocystis neurona* (Sn), *Eimeria maxima* (Em), *Eimeria praecox* (Ep), *Eimeria falciformis* (Ef), *Eimeria tenella* (Et), *Eimeria necatrix* (En), *Eimeria brunetti* (Eb), *Plasmodium falciparum* (Pf), *Plasmodium chabaudi* (Pc), *Plasmodium berghei* (Pg), *Plasmodium* *yoelii* (Py), *Plasmodium knowlesi* (Pk), *Plasmodium vivax* (Pv), *Gregarina niphandrodes* (Gn), *Cryptosporidium parvum* (Cp), *Cryptosporidium muris* (Cm), *Cryptosporidium hominis* (Ch), *Chromera velia* (Cv), *Vitrella brassicaformis* (Vb), *Paramecium tetraurelia* (Pt), *Tetrahymena thermophila* (Tt), *Saccharomyces cerevisiae* (Sc), and *Homo sapiens* (Hs). The sequences (accession numbers are shown in brackets) were *Toxoplasma gondii* PGM1 (TgPGM1) [TGME49_285980] and TgPGM2 [TGME49_318580], *Neospora canimun* A0A0F7UAD1 PGM1 (NcPGM1) [NCLIV_014450 has wrong first exon] and NcPGM2 [NCLIV_010960], *Hammondia hammondi* PGM1 (HhPGM1) [HHA_285980] and HhGPM2 [HHA_318580], *Sarcocystis neurona* PGM1 (SnPGM1) [SN3_00900660] and SnPGM2 [SN3_01700255], *Eimeria maxima* PGM1 (EmPGM1) [EMWEY_00024400], *Eimeria praecox* PGM2 [EPH_0036200], *Eimeria falciformis* PGM1 (EfPGM1) [EfaB_MINUS_40637.g2669_1], *Eimeria tenella* PGM1 (EtPGM1) [ETH_00002785], *Eimeria necatrix* PGM1 (EnPGM1) [ENH_00082780], *Eimeria brunetti* PGM1 (EbPGM1) [EBH_0076610], *Plasmodium falciparum* GGM2 (PfGGM2) [PF3D7_1012500], *Plasmodium reichenowi* GGM2 (PrGGM2) [PRCDC_1011900], *Plasmodium chabaudi* PGM2 (PcPGM2) [PCHAS_1211600], *Plasmodium berghei* PGM2 (PbPGM2) [PBANKA_1210900], *Plasmodium yoelii* PGM2 (PyPGM2) [PY17X_1214100], *Plasmodium knowlesi* PGM2 (PkPGM2) [PKNH_0812300], *Plasmodium vivax* PGM2 (PvPGM2) [PVX_094845], *Gregarina niphandrodes* PGM1 (GnPGM1) [GNI_111250], *Cryptosporidium parvum* PGM1.1 (CpPGM1.1) [cgd2_3270] and CpPGM1.2 [cgd2_3260], *Cryptosporidium muris* PGM1.1 (CmPGM1.1) [CMU_003300] and CmPGM1.2 [CMU_003310], *Cryptosporidium hominis* PGM1 (ChPGM1) [Chro.20343], *Chromera velia* PGM1 (CvPGM1) [Cvel_11076] and CvPGM2 [Cvel_22350], *Vitrella brassicaformis* PGM1 (VbPGM1) [Vbra_3443] and VbPGM2 [Vbra_19870], *Paramecium tetraurelia* GPGM1 (PtGPGM1) [AAB05649.2], *Tetrahymena thermophile* PGM2 (TtPGM1) [AAB97159.1], *Saccharomyces cerevisiae* PGM1 (ScPGM1) [CAA89741.1], and *Homo sapiens* PGM1 (HsPGM1) [AAA60080.1]. Branch colors reflect species relationships with the cyst-forming coccidia in red, non-cyst-forming coccidia in orange, *Plasmodium* spp. in purple, *Cryptosporidium* spp. in light blue, gregarines in medium blue, ciliates in dark blue, and the chromerids in green. Bar, 0.2 nucleotide substitutions per position.

To establish whether PRP1 has a universal role in exocytosis across the Apicomplexa, we first explored the conservation and phylogeny of phosphoglucomutases (PGMs) in apicomplexans and their closely related free-living relatives, the chromerids. Both PRP1 (or *Toxoplasma gondii* PGM1 [TgPGM1]) and TgPGM2 were compared to the validated PGM1/parafusins in ciliates and selected crown eukaryotes (Fig. 1). Consistent with the observation that PGM gene duplication occurred early in the emergence of the eukaryotic cell (14), these results show that all PGM1 sequences cluster together and are uniquely distinct from PGM2. The conservation of the PGMs also varied among different apicomplexan subgroups. *Toxoplasma* and the other cyst-forming coccidia (*Hammondia hammondi*, *Neospora caninum*, and *Sarcocystis neurona*) have both isoforms, while the non-cyst-forming coccidia, e.g., *Eimeria* spp., have only the PGM1 ortholog. Members of the genus *Plasmodium* have only PGM2, whereas *Cryptosporidium* spp. and the related *Gregarina niphandrodes* have PGM1; in fact, *Cryptosporidium parvum* and *Cryptosporidium muris* have two slightly different PGM1 isotypes encoded by tandem genes in the genome. We did not find any annotated PGM in the genome of *Theileria annulata*. On the other hand, chromerids (*Chromera velia* and *Vitrella brassicaformis*) have both PGMs. The ciliates *Paramecium tetraurelia* and *Tetrahymena thermophila* contain only PGM1. Overall, the phylogenetic analysis suggests that the common ancestor of the Alveolata (including apicomplexans and ciliates) contained both PGM1 and PGM2. While many lineages seem to have lost one of the PGMs, some extant lineages still maintained both the ancestral forms. *Toxoplasma*, which contains both isoforms, thus appears to be an ideal organism to study the contribution of these unique metabolic enzymes to Ca^2+^-dependent exocytosis.

### PRP1 is dispensable for the lytic cycle.

**FIG 2:**
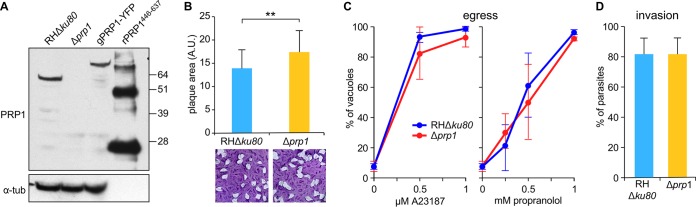
PRP1 antiserum validation and global phenotype analysis of Δ*PRP1* parasites. (A) Parental (RHΔ*ku80*), Δ*prp1*, and endogenously YFP-tagged PRP1 (gPRP1-YFP) parasites were lysed and analyzed in a Western blot using anti-PRP1 antibody. The 27-kDa shift in the molecular mass resulting from the YFP tag can be observed in gPRP1-YFP lysate. rPRP1^446-637^ represents the His_6_-tagged recombinant protein used to generate the anti-PRP1 antiserum. An antitubulin antibody (α-tub) was used as the loading control of the parasite lysate. (B, bottom) Representative images of the plaque assay performed with Δ*prp1* and parental RHΔ*ku80* parasites grown undisturbed on a host cell monolayer for 7 days. (Top) Quantification of the plaque size (in arbitrary units [A.U.]) following digital scanning of the plaque assays. Thirty plaques were counted per sample (*n* = 4). Values are means plus standard errors of the means (SEM) (error bars). Values that are significantly different (*P* < 0.001) by two-tailed *t* test indicated by the bracket and two asterisks. Statistical significance was not detected in a biological repeat of this assay (Fig. 6C). (C) Δ*prp1* and parent parasites were triggered to egress by the Ca^2+^ ionophore A23187 or propranolol to activate DGK1 and increase the PA concentration for 5 min at 37°C. For all samples, egress following stimulation is expressed as the percent egressed vacuoles of the total vacuoles observed. Values are means± standard deviations (SD) (error bars) (*n* = 30). (D) The invasion efficiency was assessed using the red-green assay with freshly lysed Δ*prp1* and parental RHΔ*ku80* parasites. The total numbers of intracellular and extracellular parasites per microscopy field were counted, and the intracellular parasites were expressed relative to the total parasites. At least 150 parasites from three random fields per sample were scored. Values are means plus SD (*n* = 3).

We first wanted to determine the essentiality of PRP1 in the tachyzoite and engineered a direct *PRP1* knockout (*PRP1*-KO) parasite line by replacing the *PRP1* gene with a hypoxanthine-xanthine-guanine phosphoribosyltransferase (HXGPRT) selectable marker (see Fig. S1 in the supplemental material). To validate the loss of protein expression, we generated a specific polyclonal antiserum against the C-terminal region of PRP1 (amino acids 446 to 637), which is unique to PRP1 and not shared with PGM2 (Fig. 2A). PRP1 is not detected in the direct KO line (Δ*prp1* line). This result immediately indicated that PRP1 is not required for completion of the lytic cycle. However, to eliminate the possibility of any growth disadvantage in the Δ*prp1* line, we performed plaque assays and compared the plaque size of the Δ*prp1* line to that of the parent line (Fig. 2B). We did observe a small but significant increase in plaque size in the absence of PRP1, although we did not detect this effect in an independent set of experiments (see below). Since earlier reports reported PRP1 involvement in microneme secretion, we examined the functioning of two essential parasitic processes relying on microneme secretion: host cell egress and invasion. We studied both highly elevated Ca^2+^-dependent and phosphatidic acid (PA)-dependent branches underlying microneme secretion (25). To induce the Ca^2+^-dependent branch, we utilized the A23187 Ca^2+^ ionophore, and to induce the PA-dependent branch, we added propranolol, which activates diacylglycerol kinase 1 (DGK1) and thereby raises the PA concentration (an overview of signaling pathways and secretagogue targets is provided in Fig. 7A). We did not observe any difference in egress efficiency compared to the RHΔ*ku80* parent line for any of the conditions tested, suggesting that micronemes are secreted sufficiently to support egress (Fig. 2C). We did not observe any difference in invasion efficiencies, indicating that secretion of the rhoptries is also likely to be unaffected in the absence of PRP1 (Fig. 2D). We directly monitored rhoptry secretion by detecting phosphorylated STAT3 (P-STAT3) accumulation in the infected-host cell nucleus, as STAT3 is phosphorylated by the rhoptry protein ROP16 (26), and we did not observe any difference in accumulation of P-STAT3 between parent and Δ*prp1* lines (Fig. S2). On the basis of these results, we conclude that PRP1 is not essential for either microneme or rhoptry secretion and is not required for completing the *Toxoplasma* lytic cycle *in vitro*.

10.1128/mSphere.00521-17.1 FIG S1  Generation and validation of the Δ*prp1* parasite line. (A) Schematic of the generation of *prp1* knockout parasites. Medium-height blue boxes indicate the 5′ and 3′ homologous regions used to replace the endogenous locus along with the endogenous promoter. Tall blue boxes represent *PRP1* exons. PCR1, PCR2, and PCR3 indicate the amplicons generated by the diagnostic PCRs (shown in panel B). (B) PCR analysis validating the replacement of the endogenous locus with the drug selection cassette (shown in panel A) using the genomic DNA of Δ*prp1* and parental RHΔ*ku80* parasites as the templates. Specific primer pairs correspond to the PCRs illustrated in panel A. M represents 1-kb DNA ladder (NEB). Download FIG S1, TIF file, 0.1 MB. Copyright © 2017 Saha et al. 2017 Saha et al. This content is distributed under the terms of the Creative Commons Attribution 4.0 International license.

10.1128/mSphere.00521-17.2 FIG S2  Absence of PRP1 does not interfere with rhoptry secretion. Rhoptry secretion was monitored by phosphorylated STAT3 (P-STAT3) accumulating in the nucleus of the infected-host cell, as STAT3 is phosphorylated by the rhoptry protein ROP16 (26). DAPI stains the nuclear material, whereas the parasite’s cytoskeleton is stained with antitubulin MAb 12G10. Download FIG S2, TIF file, 2.2 MB. Copyright © 2017 Saha et al. 2017 Saha et al. This content is distributed under the terms of the Creative Commons Attribution 4.0 International license.

### Loss of PRP1 reduces Ca^2+^-induced microneme secretion.

**FIG 3:**
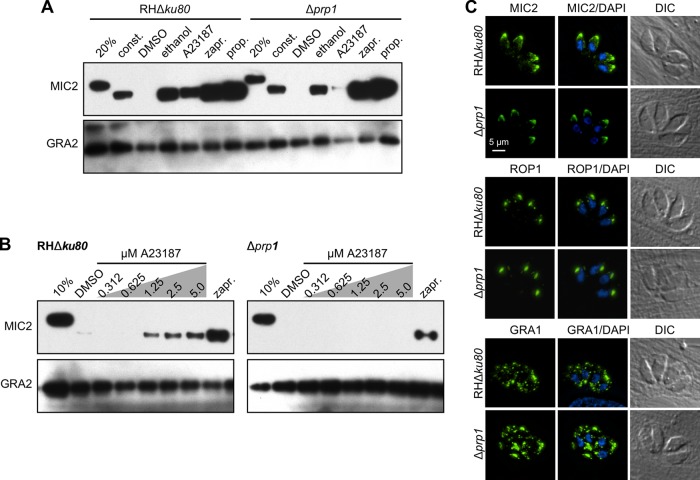
Absence of PRP1 disrupts high-Ca^2+^-trigger-induced microneme secretion in tachyzoites. (A) Representative Western blot image of the microneme secretion assay performed with Δ*prp1* and parent parasites. The secretion of the microneme protein MIC2 was used as the marker. The 20% lane contains the nonsecreted total protein lysate from 20% of the total parasites used in the secretion assay. Extracellular tachyzoites were treated with 0.25% (vol/vol) ethanol, 1.25 μM A23187, 500 μM zaprinast (zapr.), 500 μM propranolol (prop.) or DMSO as the control for 5 min at 37°C. For constitutive (const.) secretion, extracellular tachyzoites were allowed to release the protein for 1 h in the absence of a pharmacological trigger. Proteolytic processing of the secreted microneme protein can be seen as a shift in the MIC2 band. Dense granule protein GRA2 was used as a control for microneme- and Ca^2+^-independent secretion. (B) Titration of the Ca^2+^ ionophore A23187 used for triggering microneme secretion in Δ*prp1* and RHΔ*ku80* parent parasites. Extracellular tachyzoites were treated with the indicated concentrations of A23187 and zaprinast as the control for 5 min at 37°C. The 10% lane contains the nonsecreted total protein lysate from 10% of the total parasites used in the secretion assay. (C) Immunofluorescence displaying morphology of the secretory organelles in Δ*prp1* and RHΔ*ku80* parent parasites. Micronemes are shown in MIC2 panels, rhoptries are shown in ROP1 panels, and GRA1 dense granules are shown in green. DAPI marks DNA. DIC, differential interference contrast.

To assess whether the absence of PRP1 more subtly affected signaling events leading to microneme secretion, we used pharmacological triggers (secretagogues) acting on distinct steps in the signal transduction pathway. The current understanding of the signaling pathway is that protein kinase G (PKG) acts upstream of the phosphoinositide phospholipase C (PI-PLC), whose activation results in the formation of inositol triphosphate (IP_3_) and diacylglycerol (DAG) (7). Here the pathway bifurcates, as IP_3_ leads to the release of Ca^2+^ from the endoplasmic reticulum, which is further relayed by calcium-dependent protein kinases (CDPKs), whereas DAG is converted to PA, which is directly sensed by the microneme-associated sensor APH (acylated pleckstrin-homology domain-containing protein) to promote their secretion (25). We used 5-min treatments with the following. We used zaprinast to activate PKG (27, 28), ethanol to trigger PI-PLC (29), A23187 to mimic the high Ca^2+^ trigger (30, 31), and propranolol to activate DGK1, which raises the PA concentration and engages the micronemes directly (25, 32). Large increases in cytoplasmic Ca^2+^ concentration have been reported to coincide with egress and invasion, whereas intermediately elevated cytoplasmic Ca^2+^ concentrations accompany gliding motility between host cells (9, 33). Hence, we determined the level of so-called “constitutive” microneme secretion in the absence of any pharmacological secretagogues measured over 1 h, reflecting the basal level of microneme secretion during extracellular gliding (8). Secretion was assessed through the release of the proteolytically cleaved microneme protein MIC2 in the supernatant of extracellular parasites. We observed robust constitutive secretion regardless of the presence or absence of PRP1 (Fig. 3A). Even the activation of signaling events in the PA-dependent branch of the pathway and further upstream (resulting from propranolol, ethanol, or zaprinast inductions) did not result in dramatic changes in MIC2 release. However, the Δ*prp1* parasites were much less responsive to the Ca^2+^-mediated release branch of the signaling pathway triggered by A23187; the amount of released MIC2 is sharply reduced to around the detection limit. Thus, PRP1 plays a role only in the Ca^2+^-dependent signal transduction pathway in microneme secretion and does not play a role in the PA-mediated branch contributing to secretion.

To differentiate whether the loss of the high Ca^2+^ response is the result of an overall deficiency or the result of reduced sensitivity, we titrated the amount of A23187 in the microneme secretion assay (Fig. 3B). Compared to wild-type parasites that secrete MIC2 at A23187 concentrations as low as 1.25 µM and reached saturation at 2.5 µM, Δ*prp1* mutants never display any secretion even in the presence of 5 µM A23187. The defect in Δ*prp1* parasites therefore appears to be only in their ability in response to large increases in the Ca^2+^ concentration contributing to microneme secretion.

Since PFUS in *Paramecium* has been shown to affect secretory organelle formation (19), we reasoned that possible mistrafficking of the microneme proteins in the Δ*prp1* mutants could also explain its secretion defect. We tested this, as well as trafficking to the other secretory organelles, by fluorescence imaging using MIC2 antibody alongside rhoptry- and dense-granule-specific antisera (Fig. 3C). We observed no accumulation of MIC2 protein along the secretory pathway, and the morphology of all secretory organelles, including the micronemes, was normal. Thus, the loss of PRP1 does not affect organellogenesis or protein trafficking to the micronemes but results in the inability to enhance microneme secretion in response to high Ca^2+^ concentrations.

### Where does PRP1 localize? 

PRP1 was previously shown to localize to the most apical (radial) micronemes, and upon triggering microneme secretion with ethanol transitions to the cytoplasm (22, 23). We sought to confirm this observation with our specific PRP1 antiserum. The polyclonal antiserum generated recognized a single band around 64 kDa in wild-type parasites, which is close to the predicted size of 70 kDa for the PRP1 protein (Fig. 2A). No band was detected in the Δ*prp1* line, which clearly illustrated the high specificity of our antiserum. Using previously reported 4% paraformaldehyde (PFA) fixation, we did not observe any specific signal except a random spotty anti-PRP1 pattern that appeared identical in wild-type and Δ*prp1* parasites (Fig. S3A). Methanol fixation resulted in a cytoplasmic signal that was absent in Δ*prp1* parasites, suggesting signal specificity. Since our localization contradicts previous observations and PRP1 has been reported to shuttle between microneme association and cytoplasmic presence, we decided to fractionate live, wild-type parasites in membrane and cytosolic fractions by differential centrifugation (Fig. S3B). We used CDPK1 as a cytosolic marker (34) and MIC2 antiserum to probe for micronemal proteins. These data show that a small fraction of PRP1 is present on vesicular structures (P1 in Fig. S3B) and potentially cofractionates with the micronemes (S2 in Fig. S3B; we note that MIC2 appears proteolytically processed and that this fraction therefore likely represents secreted MIC2 rather than intact micronemal contents). Most PRP1 was equally distributed in soluble (S2) and membrane (P2) fractions, suggesting equal amounts of cytosolic and membrane association. Thus, these data indicate that in *Toxoplasma*, PRP1 is present in both soluble and membrane-associated forms, the latter of which is not appreciated by immunofluorescence imaging. Moreover, since our antiserum is incompatible with PFA fixation, a direct comparison to previous PRP1 localization reports is not possible (22, 23).

10.1128/mSphere.00521-17.3 FIG S3  Subcellular localization of PRP1 by antiserum. (A) PRP1 antiserum was used to probe methanol (MetOH)- or 4% paraformaldehyde (PFA)-fixed wild-type (RHΔ*ku80*) and Δ*prp1* parasites, intracellular or extracellular, as indicated. IMC3 antiserum was used as a control for the cortical cytoskeleton. DAPI stains DNA. Note that PFA fixation either destroys the epitope recognized by the PRP1 antiserum or destroys the structure to which PRP1 localizes. (B) Fractionation of wild-type parasites shows that PRP1 is present in both a membrane-associated and non-membrane-associated, soluble fraction. (Top) Flowchart of fractionation strategy. (Bottom) Western blots of fractions probed with antisera as indicated. The sizes of the proteins are as follows: PRP1, 64 kDa; CDPK1, 65 kDa; MIC2, 87 kDa. Download FIG S3, TIF file, 1.4 MB. Copyright © 2017 Saha et al. 2017 Saha et al. This content is distributed under the terms of the Creative Commons Attribution 4.0 International license.

**FIG 4:**
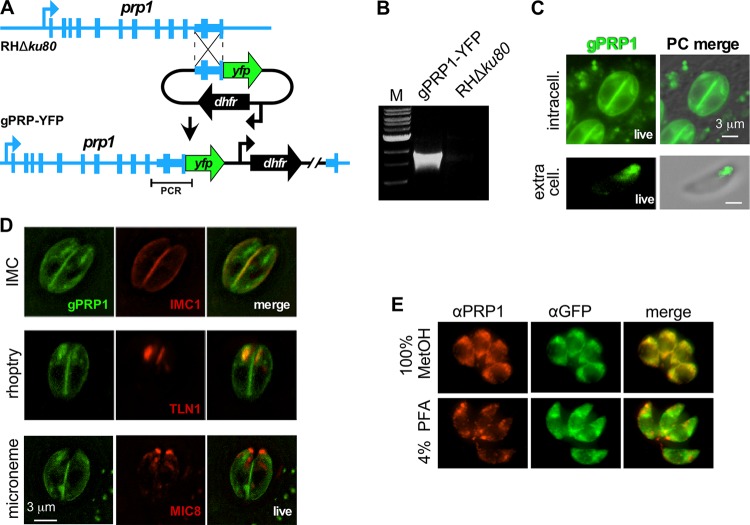
Subcellular localization of PRP1 through endogenous tagging. (A) Schematic representation of generating C-terminal endogenously YFP-tagged gPRP1-YFP parasites by single homologous recombination into the RHΔ*ku80* parent line. (B) PCR validation of the gPRP1-YFP genotype using the primer pair shown in panel A. Lane M contains 1-kb DNA ladder (New England Biolabs). (C) Live imaging of gPRP1-YFP parasites under intracellular and extracellular conditions as indicated. PC, phase contrast. (D) Live imaging of gPRP1-YFP parasites cotransfected with markers for the IMC (IMC1-mCherry), rhoptries (TLN1-mCherry), and micronemes (MIC8-mCherry). (E) Representative images of intracellular gPRP1-YFP parasites fixed using either 100% methanol (MetOH) or 4% paraformaldehyde (PFA) stained with anti-PRP1 (αPRP1) and anti-GFP (αGFP) antisera as indicated. Note that PFA fixation destroys the costaining of GFP and PRP1 and thus destroys the PRP1 epitope(s) recognized by the specific antiserum.

To further elucidate the PRP1 localization, we tagged the endogenous locus at the C terminus with a yellow fluorescent protein (YFP) (endogenously YFP-tagged PRP1 [gPRP1-YFP]; Fig. 2A and 4A and B). Live imaging of intracellular parasites revealed that YFP localized to the cortex as well as to an extended apical structure, the latter of which was reduced in extracellular parasites (Fig. 4C). Cotransfections targeting red fluorescent fusion proteins to the cortical inner membrane complex (IMC) cytoskeleton (IMC1), rhoptries (TLN1), or micronemes (MIC8) showed that the cortical gPRP1-YFP signal colocalized with the IMC, whereas the elongated apical signal overlaid the rhoptries (Fig. 4D). Surprisingly, we observed no colocalization with the micronemes under any condition tested. We further used this YFP-tagged PRP1 line to validate our anti-PRP1 antiserum upon methanol and PFA fixation through costaining with an anti-green fluorescent protein (anti-GFP) antiserum recognizing YFP. We again observed that PFA fixation disrupted anti-PRP1 epitope recognition, since there was no overlap with the evenly distributed anti-GFP signal. Although we observed an overlap in cytoplasmic GFP and PRP1 localization upon methanol fixation, this pattern differed from the IMC and rhoptry localization evident in live parasites, suggesting a fixation artifact. We therefore concluded that endogenously tagged PRP1 localizes to the IMC and rhoptries and that fixation either induces artifacts and/or is incompatible with our antiserum.

### PGM2 also functions in microneme secretion.

**FIG 5:**
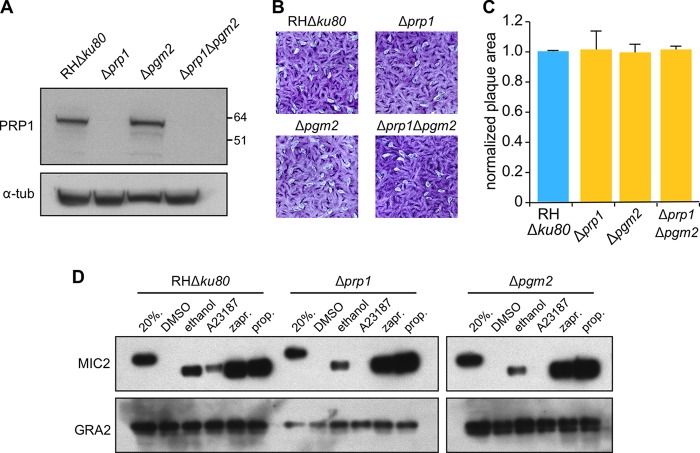
PGM2 is also required for Ca^2+^ trigger-induced microneme secretion and does not complement the loss of PRP1. (A) Diagnostic Western blot to validate the presence or absence of PRP1 protein in the Δ*pgm2* parasite lines generated in the RHΔ*ku80* or Δ*prp1* background, respectively. The parental RHΔ*ku80*, Δ*prp1* and Δ*pgm2* single mutants and Δ*prp1* Δ*pgm2* double mutant are shown. Antitubulin antibody was used as a loading control. Numbers to the left of the blot represent the molecular weights (in thousands) of the proteins. (B) Representative images of plaque assays performed with parasite lines as indicated, grown undisturbed on a host cell monolayer for 7 days. (C) Quantification of the plaque size following digital scanning of the plaque assays. The plaque sizes in the biological replicate experiment were normalized to those of the parent line. A total of 30 plaques were counted per sample (*n* = 3). Values are means ± SEM. No significant differences were detected using the two-tailed *t* test. (D) Representative Western blot image of the MIC2 microneme secretion assay. The 20%. lanes contain the nonsecreted total protein from 20% of the total parasites used for the secretion assay. Extracellular tachyzoites were treated with DMSO control, ethanol, A23187, zaprinast (zapr.), or propranolol (prop.) for 5 min at 37°C or assayed for constitutive (const.) secretion for 1 h. Dense granule protein GRA2 was used as a control for microneme- and Ca^2+^-independent secretion.

To ensure that the phenotypic effects observed in the Δ*prp1* mutants are specific to *prp1* deletion and not due to any potential functional compensation by the PGM2 paralog, we also deleted this gene. We replaced the *pgm2* locus with a drug resistance cassette mediated by a clustered regularly interspaced short palindromic repeat (CRISPR)/Cas9-induced double-strand break, both in parent (RHΔ*ku80*) and Δ*prp1* parasites (Fig. 5A and Fig. S4). Plaque assays showed that both the single (Δ*prp1* and Δ*pgm2*) and double (Δ*prp1* Δ*pgm2*) knockout lines were viable and did not show any significant difference in plaque formation competence (Fig. 5B and C). To specifically understand the effect of deleting *pgm2* and determine its role in microneme secretion, we performed secretion assays alongside the Δ*prp1* knockout line and the parental strain (Fig. 5D). Interestingly, the deletion of *pgm2* demonstrated microneme secretion defects similar to those observed in the absence of the *prp1* gene: A23187-induced MIC2 secretion was not detected, whereas the release upon zaprinast and propranolol triggers was unchanged. Thus, our data suggest that PRP1 and PGM2 act similarly in the microneme secretion pathway, thereby eliminating any putatively compensatory function between the two proteins.

10.1128/mSphere.00521-17.4 FIG S4  Generation of *pgm2* knockout parasite lines. (A) Schematic representation of generating *pgm2* knockouts by double homologous recombination into the RHΔ*ku80* or Δ*prp1* parasite as the parent line. Blue boxes indicate the homologous regions used to replace the endogenous locus. The light blue boxes indicate the exons in the genomic locus of *pgm2*. PCR1 to PCR5 indicate the diagnostic PCRs (shown in panel B). (B) Diagnostic PCRs validating the replacement of the *pgm2* locus with the *DHFR* cassette in both the RHΔ*ku80* and Δ*prp1* background. M represents 1-kb DNA ladder (NEB). Download FIG S4, TIF file, 0.4 MB. Copyright © 2017 Saha et al. 2017 Saha et al. This content is distributed under the terms of the Creative Commons Attribution 4.0 International license.

10.1128/mSphere.00521-17.5 FIG S5  PRP1 does not interact with PGM2 measured by co-IP. RHΔ*ku80* parasites expressing Myc2-PGM2 or untransfected control parasites were immunoprecipitated with anti-Myc beads and Western blots (WB) probed with either Myc or PRP1 antisera as indicated. No interaction between Myc2-PGM2 and PRP1 is detected. Lanes: CL, cleared lysate; FT, flowthrough; W1, wash 1; W2, wash 2; W3, wash 3; EL, eluate. Download FIG S5, TIF file, 0.3 MB. Copyright © 2017 Saha et al. 2017 Saha et al. This content is distributed under the terms of the Creative Commons Attribution 4.0 International license.

The shared phenotype between Δ*prp1* and Δ*pgm2* lines suggested either a sequential role of the proteins in the same pathway or an interaction between the two proteins. Proteins within the alpha-d-phosphohexomutase superfamily to which the PGMs belong in general form dimers (e.g., *Paramecium* parafusin is a symmetric dimer [35]), whereas there is an example of a heterodimer of allelic variants (36). As such, we explored the possibility of PRP1/PGM2 heterodimers in *Toxoplasma* tachyzoites. To this end, we generated a parasite line expressing a tandem Myc epitope fusion of PGM2 (Myc2-PGM2). Subsequently, we performed coimmunoprecipitation with Myc epitope recognizing 9E10 antibodies and probed with our PRP1-specific antiserum. We readily pulled down the Myc2-PGM2 in this assay; however, we were unable to detect PRP1 in the same fraction (Fig. S5). Therefore, these data do not support a physical association between PRP1 and PGM2 under the conditions tested.

### Neither PRP1 nor PGM2 is required for acute virulence.

**FIG 6:**
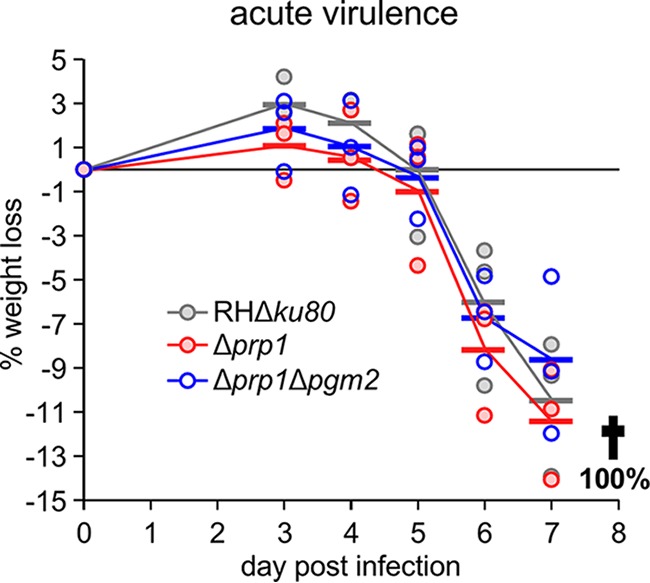
Loss of the PRP1/PGM2 complex does not affect acute virulence in mice. Acute virulence in C57BL/6J mice was assessed by intraperitoneal (i.p.) infection of 1,000 tachyzoites of the indicated parasite lines. Weight changes relative to day 0 are shown for each mouse. Each symbol shows the weight change for an individual mouse. Average values for the groups are shown by the horizontal bars. The black line represents the average value for all groups. All mice in each group either died or were euthanized at day 8 postinfection (p.i.).

Across the Ca^2+^-dependent exocytosis function of the PGM orthologs in *Toxoplasma*, we observe a role in microneme secretion but no effect on the plaque-forming capacity of tachyzoites *in vitro*. However, the data do not eliminate the fact that the PGM orthologs are essential during animal infection, where the environment is much more complex. Germane to this point, Ca^2+^ has been shown to be critical for evading the egress-mediated innate immune response during acute mouse infection (37). It is conceivable that this escape response needs to be fast and could rely on large increases in intracellular Ca^2+^. To test this model, we assessed the acute virulence of the Δ*prp1* and Δ*prp1* Δ*pgm2* mutants through mouse infections. We observed no difference in the progression of infections between the mutants or RHΔ*ku80* parent line (Fig. 6). Weight change patterns were comparable for all infected mice, and all mice either died or needed euthanasia on day eight postinfection. Thus, these data indicate that the PGM orthologs are not only dispensable *in vitro* but also *in vivo* during an acute infection.

## DISCUSSION

**FIG 7:**
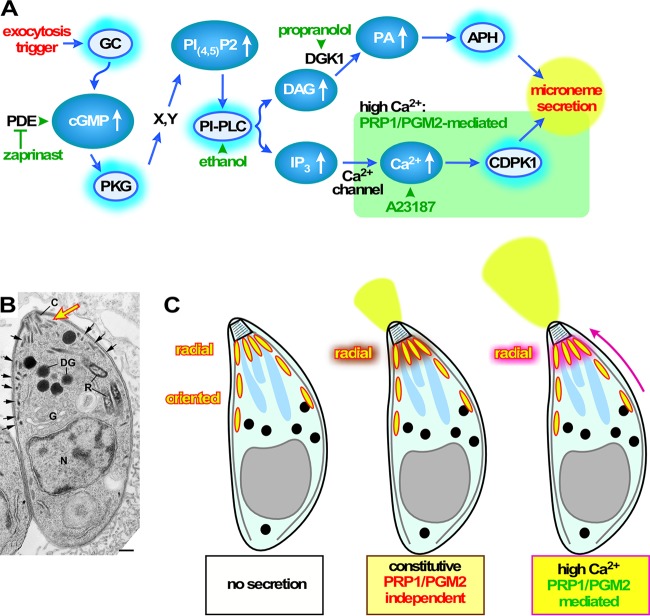
Schematic overviews of signaling and role of PRP1/PGM2 in microneme exocytosis. (A) Schematic overviews of signaling pathways and action of secretagogues. GC, guanylate cyclase; PDE, phosphodiesterase; PKG, protein kinase G; PI-PLC, phosphoinositide phospholipase C; DGK1, diacylglycerol kinase 1; APH, acylated pleckstrin-homology domain-containing protein; CDPK1, calcium-dependent protein kinase 1. X and Y represent proteins of unknown identity and number. Activated proteins are represented by a teal halo. (B) Electron microscopy image of a tachyzoite. Radial micronemes arranged under the conoid (c) are indicated by the large yellow arrow; small black arrows point to oriented micronemes. DG, dense granules; R, rhoptry; DG, dense granules; G, Golgi apparatus; N, nucleus in microneme secretion. Bar, 200 μm. (Image republished from reference 73 with permission of the publisher.) (C) Schematic representation of the process mediated by PRP1 and PGM2. The pink arrow indicates fast replenishment of the oriented micronemes (yellow with red border) to boost the radial micronemes, which are secreted first, resulting in a boost of secreted micronemes. Dense granules are black, and rhoptries are blue.

Ca^2+^-mediated exocytosis is a key event required for successful host cell invasion of apicomplexan parasites. *Toxoplasma* PRP1 was associated with this process based on localization studies in *Toxoplasma* and heterologous functional studies in the ciliate *Paramecium* (23). Here we directly assessed the role of PRP1 in *Toxoplasma* by deleting the gene from the parasite, which surprisingly resulted in a nonlethal phenotype both *in vitro* and *in vivo*. We did, however, identify a defect in the release of micronemes triggered by elevated Ca^2+^ concentrations (insights are summarized in Fig. 7). Our detailed analysis of the *prp1* deletion mutant revealed that the morphology of the secretory organelles and cytoskeletal inner membrane complex (IMC) remain normal in Δ*prp1* parasites. Furthermore, we demonstrated that invasion and egress, which are both reliant on successful Ca^2+^-mediated exocytosis, are independent of PRP1.

Since PRP1 was hypothesized to play a role in microneme secretion, we determined the dynamics of *in vitro* microneme secretion under different conditions and stimulation treatments. Our work demonstrates that the deletion of *prp1* affects only microneme secretion bursts triggered by a high Ca^2+^ concentration through stimulation with the Ca^2+^ ionophore A23187. Titration of A23187 demonstrated that PRP1 does not act as an amplifier of low Ca^2+^, as the secretion boost in response to high Ca^2+^ was abolished regardless of the A23187 concentration. Overall, these data indicate that PRP1 is essential to translate a high Ca^2+^ trigger into enhanced microneme secretion (Fig. 7B), and thereby adds a new layer of complexity to the signaling pathways underlying Ca^2+^-mediated secretion that is, to our knowledge, detected here for the first time.

High concentrations of intracellular Ca^2+^ are known to occur during invasion and egress (8, 9, 33), which correlate with increased levels of microneme secretion. However, since we observed no apparent growth defect and maybe even a growth advantage of the Δ*prp1* parasites, the physiological function of elevated microneme secretion is currently not clear. Constitutive microneme secretion in the absence of pharmacological stimuli is comparable between the Δ*prp1* and wild-type parasites. This suggests that a basal level of microneme secretion is sufficient to complete the lytic cycle of the parasite. However, we cannot exclude the possibility that the ability to respond to high Ca^2+^ fluxes and the subsequent microneme secretion response might be critical during other developmental stages of the *Toxoplasma* life cycle.

The PGM mutants allowed us to dissect the contribution of different branches of the signaling pathway toward microneme secretion through stimulation with targeted pharmacological secretagogues (Fig. 7A). Upstream stimulation of the pathway with PKG-activating zaprinast leads to robust microneme secretion in these mutants. In *Plasmodium*, numerous protein substrates have been reported to be PKG substrates (38), which likely have many parallels in *Toxoplasma*. In *Plasmodium*, PKG controls phosphoinositide biosynthesis through an unmapped mechanism, and the inhibition of PKG blocks hydrolysis of phosphatidylinositol 4,5-bisphosphate [PI_(4,5)_P_2_] (38). PI_(4,5)_P_2_ is the substrate for PI-PLC, which in turn forms IP_3_ and DAG. IP_3_ is associated with inducing the increase in cytoplasmic Ca^2+^ concentration, resulting in microneme secretion (39, 40), whereas DAG is converted into PA, which is directly sensed by the micronemes to promote their secretion (25). These results show that the phosphoinositide metabolism mediated by PKG activity is a major, but not the only, requirement for a complete microneme secretion response and thus implies the presence of other contributing PKG substrates. Excluding the contribution of the Ca^2+^-mediated branch by specifically triggering PA production through propranolol stimulation releases all brakes on microneme secretion (25) and leads to higher secretion than PKG stimulation regardless of the presence of PRP1. This indicates that very high PA levels override the requirement of substrates except those affecting phosphoinositide metabolism for PKG. Thus, our work provides evidence for PKG substrates contributing to the microneme secretion response. In ciliates, PFUS is phosphorylated by casein kinase II (CKII) and PKG (41), while during exocytosis the protein undergoes dephosphorylation by the protein phosphatase calcineurin (CN) (17, 42–44). The dephosphorylation by CN results in dissociation of PFUS from the ciliate secreting vesicles, while the released PFUS reassociates with nascent vesicles upon phosphorylation. It is conceivable that PRP1 phosphorylation by PKG is required for microneme secretion, but our data on *Toxoplasma* CN demonstrate that this is not the key activity of CN, as microneme secretion remains unaffected upon CN depletion (24).

Using a peptide antibody against PFUS, which cross-reacts with PRP1, the latter was reported to localize to the most apical micronemes from which it releases in a phosphorylation state-dependent fashion upon secretion (22). These most apical micronemes are known as the micronemes due to their tightly packed organization around the conoid at the very apical end of the parasite (45) (Fig. 7B). These micronemes most likely represent a “readily releasable secretory vesicle pool” of predocked micronemes as have been reported for other secretory cells that can be released immediately upon the appropriate triggers (46, 47). For subsequent and continuous secretion replenishment, micronemes lying further basally along the subpellicular microtubules in the cell (the so-called “oriented” micronemes along the microtubules [48]) have to be transported to the apical end. Since the first step already occurred in the parasites we tested when they egressed from the host cell, it is the fast replenishment of releasable micronemes that appears to require PRP1/PGM2 (Fig. 7C, right schematic representation). Thus, in this model, PRP1 controls the release of micronemes in doses. The IMC and rhoptry localization we observe with YFP-fused PRP1 in intracellular parasites and partial release from the rhoptries would be compatible to some extent with this model: PRP1 is there if the micronemes need to be activated for transport along the IMC. Notably, protein kinase A (PKA) signaling contribution to microneme secretion was recently reported to also be localized on the IMC (49), and therefore may serve as a signaling scaffold.

Although our PRP1 antiserum was highly specific in Western blots, unfortunately, it was incompatible with indirect immunofluorescence assay (IFA) fixation methods, thereby restraining us from repeating the reported experiments. Membrane-associated proteins are notoriously challenging to localize subcellularly due to fixation artifacts (50), which we also found to be the case. However, we were able to confirm that PRP1 is present in soluble and membrane-associated forms in extracellular parasites, which is consistent with the previously presented model (51). In our opinion, the YFP fusion data are the best reflection of the natural PRP1 localization.

To ensure that the phenotypic effects observed in the Δ*prp1* mutants are specific to PRP1 deletion and not due to potential functional compensation by the PGM2 paralog, we also removed this gene and even made a double knockout mutant. The observation that deletion of either one of the PGM genes simultaneously results in the same microneme secretion defect upon high Ca^2+^ flux sheds a completely new light on the specific role of PRP1 in Ca^2+^ signaling: PRP1 is not the sole protein playing this role in this function, as it extends to the other PGM expressed in *Toxoplasma*. This observation might explain why the conservation of either ortholog in the various Apicomplexa is a mishmash (Fig. 1), as either ortholog could function in Ca^2+^-dependent secretion. However, the fact that individual gene deletions in *Toxoplasma* resulted in the same phenotype suggests a functional interaction between PRP1 and PGM2 and hence a likely selective evolutionary pressure on preserving both. By coimmunoprecipitation (co-IP), we did not detect any physical interaction. Alternatively, the interaction might be transient upon high Ca^2+^ levels, which is much harder to capture.

In addition, our data show that *Toxoplasma* tachyzoites can survive without dedicated phosphoglucomutase. Similar observations have been made in other organisms. For example, knockdown of PGM in *Saccharomyces cerevisiae* (52–54) or depletion of the single PGM in *Paramecium* and *Tetrahymena* (19, 20) demonstrated no apparent effect on the growth of these organisms. In fact, there are even organisms without a dedicated PGM such as *Trypanosoma brucei* (55). In addition, we showed that the PFUS-related PGM orthologs versus the exclusive PGM enzymes are not at all uniformly conserved across the Apicomplexa (Fig. 1), which further supports a flexible role for these proteins across organisms. It has been established in several systems that alpha-d-phosphohexomutase superfamily enzymes can also have phosphoglucomutase activity, such as phosphomannomutase (PMM) and phospho-*N*-acetylglucosamine mutase (PAGM). Indeed, *Toxoplasma* has a PMM (accession no. TGME49_239710) and a PAGM (TGME49_264650), and the amino acids involved in sugar binding are conserved between the *Toxoplasma* and *T. brucei* PMMs (55). These insights could therefore explain why the deletion of both *Toxoplasma* PGMs has no apparent effect on energy metabolism. However, it is conceivable that glycogen storage under the conditions we tested is not required and might become more relevant during bradyzoite differentiation, when storage compartments become more defined (56).

In conclusion, we show that PRP1 and PGM2 function in translating a large increase in the cytoplasmic Ca^2+^ concentration into a burst of microneme secretion. The gene knockout lines also confirmed the bifurcation in the Ca^2+^- and PA-mediated control of microneme secretion downstream of PI-PLC activity. The constitutive level of microneme secretion is independent of PRP1 or PGM2 and sufficient to support the parasite through all steps of the lytic cycle both *in vitro* and *in vivo*. We further conclude that PRP1 and PGM2 functions control microneme secretion at a level not previously identified. Considering the mosaic phylogeny pattern of PGM ortholog conservation across the Apicomplexa, our data thus suggest that this control mechanism of the microneme secretion pathways could be widespread among the eukaryotes.

## MATERIALS AND METHODS

### Parasites and host cells.

*Toxoplasma* tachyzoites were maintained in human foreskin fibroblasts (HFF) as previously described (57). The host cells were maintained in Dulbecco modified Eagle medium (DMEM) containing 10% serum. *Toxoplasma* strain RHΔ*ku80*Δ*HX* (58) was used as the basis for all mutants in this study. Stable transgenics were obtained by selection with 1 µM pyrimethamine, 25 µg/ml mycophenolic acid (MPA) combined with 50 µg/ml xanthine or 20 µM chloramphenicol and cloned by limiting dilutions.

### Plasmids.

All primer sequences are provided in Table S1 in the supplemental material. For tagging the endogenous PRP1, 1.5-kb genomic DNA upstream of the stop codon was PCR amplified using primer pair PRP1-LIC-F and PRP1-LIC-R (PRP1-LIC-F/R) (LIC stands for ligation-independent cloning, F stands for forward, and R stands for reverse) and cloned by ligation-independent cloning into plasmid pYFP-LIC-DHFR (YFP stands for yellow fluorescent protein) (kindly provided by Vern Carruthers, University of Michigan) (58). The plasmid was linearized with NcoI prior to transfection.

10.1128/mSphere.00521-17.6 TABLE S1  Sequences of primers used in this study. Download TABLE S1, PDF file, 0.1 MB. Copyright © 2017 Saha et al. 2017 Saha et al. This content is distributed under the terms of the Creative Commons Attribution 4.0 International license.

For the generation of Δ*prp1* plasmid, we deleted the LoxP flanked tubulin promoter and YFP cassette from the p5RT70-loxP-KillerRed-loxP-YFP/HX plasmid (59) (kindly provided by Markus Meissner, University of Glasgow) by ApaI/NotI digestion, blunting the ends, and religation. The 2-kb flanks for double homologous recombination (HR) were PCR amplified from RHΔ*hx* genomic DNA using primer pairs 5′PRP1-KpnI-F/R for the 5′ flank located 1.3 kb upstream of the ATG start codon and 3′PRP-SacI-F/R for the 3′ flank downstream of the translation stop. The 5′ flank was inserted into p5RT70--/HX using KpnI, and independently, the 3′ flank was inserted separately in parallel into p5RT70--/HX using SacI; the two plasmids were conjoined using ScaI/NotI to make the final double homologous recombination plasmid. The plasmid was linearized with ScaI prior to transfection.

Plasmid ptub-Myc2-PGM2/sagCAT (tub stands for tubulin, and CAT stands for chloramphenicol acetyltransferase) was cloned by PCR amplifying the PGM2 coding sequence (CDS) from cDNA using primer pair PGM2-AvrII-F and PGM2-EcoRV-R and was cloned by AvrII/EcoRV digestion into the ptub-Myc2-GAPDH1/CAT (GAPDH stands for glyceraldehyde-3-phosphate dehydrogenase) (60).

### Generation of PGM2 knockout.

To generate Δ*pgm2* parasites, two CRISPR/Cas9 plasmids targeting Cas9 to the *pgm2* genomic locus were designed using the primer pairs 5PGM2-dKO-s/as (dKO stands for double knockout, s stands for sense, and as stands for antisense) and 3PGM2-dKO-s/as upstream of AUG and downstream of the stop codon, respectively. The specificity of the guide RNA was tested as previously described (61). The dihydrofolate reductase (DHFR) drug selectable cassette along with 20 nucleotides of homologous region flanking each end, which corresponds to upstream and downstream of the genomic locus, was PCR amplified. Forty micrograms of each pU6-5’PGM2/3’PGM2-Cas9 plasmid was cotransfected with 40 µg of the PCR product into RHΔ*ku80*Δ*hx* or RHΔ*ku80ΔhxΔprp1* parasites to generate Δ*pgm2* parasites.

### Immunofluorescence assays and live fluorescence microscopy.

Live microscopy was performed on intracellular parasites grown overnight in six-well plates containing coverslips confluent with HFF cells. For extracellular localization, freshly lysed parasites were filtered, pelleted, and resuspended in phosphate-buffered saline (PBS). Thereafter, parasites were added to poly-l-lysine-coated coverslips and allowed to incubate for 30 min at 4°C prior to imaging. Colocalization studies with live gPRP1-YFP parasites was performed following transient cotransfection with the following plasmids: tub-IMC1mCherryRFP/sagCAT (RFP stands for red fluorescent protein) (62), TLN1(1-58)mCherryRFP/HPT (rhoptry marker) (63) (kindly provided by Peter Bradley, University of California at Los Angeles [UCLA]), and pTgMIC8-TgMIC8mycmCherryFP-nosel (pG53) (64) (kindly provided by Markus Meissner, University of Glasgow).

Indirect immunofluorescence assay was performed on intracellular parasites grown overnight in six-well plates containing coverslips confluent with HFF cells fixed with 100% methanol (unless stated otherwise) using the following primary antisera: rabbit anti-GFP (diluted 1:500) (Torrey Pines Biolabs), anti-MIC2 monoclonal antibody (MAb) 6D10 (1:8,000; kindly provided by David Sibley, Washington University in St. Louis [65]), rabbit anti-MIC2 (1:8,000; kindly provided by David Sibley, Washington University in St. Louis [66]), mouse anti-ROP1 (1:1,000; kindly provided by Peter Bradley, UCLA [67]), anti-GRA1 (1:20,000; kindly provided by Marie-France Cesbron-Delauw, Université Grenoble, France [68]), rat anti-IMC3 (1:2,000) (62), and guinea pig anti-PRP1 (1:1,000). Alexa Fluor 488 (A488)- or A594-conjugated goat anti-mouse, anti-rabbit, anti-rat, or anti-guinea pig were used as secondary antibodies (1:500; Invitrogen). DNA was stained with 4′,6-diamidino-2-phenylindole (DAPI). Fixation and staining of phosphorylated STAT3 (P-STAT3) (1:120) (catalog no. 9145P; Cell Signaling) was performed exactly as described previously (69). A Zeiss Axiovert 200 M wide-field fluorescence microscope equipped with a α-Plan-Fluar 100×/1.3-numerical-aperture (NA) and 100×/1.45-NA oil objectives and a Hamamatsu C4742-95 charge-coupled-device (CCD) camera were used to collect images, which were deconvolved and adjusted for phase contrast using Volocity software (Improvision/PerkinElmer).

### Generation of specific PRP1 antiserum.

To generate N-terminal His_6_-tagged fusion protein, 580 bp from the cDNA of PRP1 (corresponding to amino acids 446 to 637 in the C-terminal region of the protein) were PCR amplified using the primers aPRP1-LIC-F/R and cloned into the pAVA0421 plasmid using ligation-independent cloning (LIC) (70). The fusion protein was expressed in *Escherichia coli* BL21 using 1 mM isopropyl-β-d-thiogalactopyranoside (IPTG) overnight at 37°C and purified under denaturing condition over nickel-nitrilotriacetic acid (Ni-NTA) agarose (Invitrogen). Polyclonal antiserum was generated by immunizing guinea pigs (Covance, Denver, PA).

### Western blotting.

Following SDS-PAGE, polyvinylidene difluoride (PVDF) membrane blots were probed with mouse anti-IMC1 (diluted 1:2,000; kindly provided by Gary Ward, University of Vermont), guinea pig anti-PRP1 (1:10,000), mouse monoclonal anti-GRA1 (1:20,000), or CDPK1 nanobody (1 μg/ml, a kind gift of Sebastian Lourido, Whitehead Institute [71]) followed by probing with horseradish peroxidase (HRP)-conjugated anti-mouse (1:10,000), anti-guinea pig antibody (1:3,000, Santa Cruz Biotech), or in case of the nanobody, anti-penta-His antibody (1:10,000; Qiagen) and detection of signal by chemiluminescent HRP substrate (Millipore).

### Fractionation.

Subcellular fractionation was performed as described previously (60). Essentially, extracellular parasites were lysed by 5-min freezing of the pellet in liquid nitrogen and resuspension at 37°C in hypotonic buffer (10 mM Tris [pH 7.8] and 5 mM NaCl) followed by additional lyses by 40 Dounce homogenizer strokes. The lysate was centrifuged at low speed (1,000 × *g* for 15 min). The pellet was resuspended in an equal volume of resuspension buffer (100 mM Tris [pH 7.8] and 150 mM NaCl) and centrifuged at high speed (100,000 × *g* for 60 min). For SDS-PAGE, pellets were resuspended in an equal volume of SDS-PAGE loading buffer, and corresponding amounts were analyzed by Western blotting.

### Plaque assay.

T12.5 culture flasks or six-well plates confluent with HFF cells were inoculated with 100 parasites of choice and grown for 7 days. Following incubation, the monolayer was fixed with 100% ethanol for 10 min and stained with crystal violet (57). Plaque sizes were quantitated using ImageJ-win32 software (72).

### Microneme secretion assay.

Microneme secretion assays using the MIC2 protein were performed as described previously (30). Briefly, freshly lysed parasites were pelleted, washed, and resuspended to 2 × 10^8^/ml parasites in DMEM/FBS (DMEM containing 20 mM HEPES [pH 7.0] and 3% [wt/vol] fetal bovine serum [FBS]). Parasites (2 × 10^7^) were added to each well of a 96-well plate, and secretion was induced by the following secretagogues and concentrations unless concentrations are specifically indicated otherwise: 1.25 μM A23187, 0.25% (vol/vol) ethanol, 500 μM zaprinast, 500 μM propranolol or dimethyl sulfoxide (DMSO) control for 5 min at 37°C. For constitutive microneme secretion, we incubated parasites at 37°C for 60 min in the absence of secretagogue. Microneme secretion was assessed in the supernatant with mouse monoclonal anti-MIC2 6D10 (1:8,000). Ca^2+^-independent constitutive secretion of the dense granules was determined using mouse monoclonal anti-GRA1 (1:20,000) or rabbit anti-GRA2 antiserum (1:10,000).

### Invasion assay.

The red-green invasion assay was performed as previously published (73, 74) with modifications. Tachyzoites (2.5 × 10^5 ^to 3 × 10^5^) were added to host cells grown in 96-well black/optical bottom plates, centrifuged (500 rpm, 3 min, room temperature [RT]), and allowed to invade the host cells for 1 h at 37°C. Noninvading extracellular parasites were detected using A594-conjugated anti-SAG1/P30-T41E5 antibodies (1:500; kindly provided by Jean François Dubremetz, University of Montpellier, France [75]) or anti-SAG1 MAb DG52 (kindly provided by Jeroen Saeij, Massachusetts Institute of Technology [76]). Following 16% formaldehyde/8% glutaraldehyde fixation and permeabilization using 0.25% Triton X-100, the parasites were incubated with A488-conjugated anti-SAG1/P30 antibody to visualize both invaded and noninvaded parasites. Each incubation with antibody was followed by three washes with HH buffer (Hanks’ balanced salt solution containing 1 mM HEPES [pH 7.0]). Cytochalasin D-treated wild-type parasites were used as a negative control. Images were taken using EVOS FL cell imaging system (Life Technologies).

### Egress assay.

The egress assay was performed as described previously (73, 77). Six-well plates containing coverslips confluent with HFF cells were infected with 6 × 10^4^ RHΔ*ku80* or Δ*prp1* parasites expressing cytoplasmic YFP (78) and grown for 30 to 35 h. Egress was triggered by treatment with A23187 or propranolol at the concentration indicated using DMSO as a control at 37°C for 5 min, followed by 100% methanol fixation for 10 min at RT. Intact vacuoles were counted for at least 10 fields in two independent experiments.

### Coimmunoprecipitation.

Coimmunoprecipitation basically followed published procedures (79). Briefly, extracellular parasite pellets were subjected to one freeze-thaw cycle and lysed in lysis buffer (1× PBS, 0.25% NP-40, 400 mM NaCl, 250 U/ml Benzonase [Novagen], mammalian protease inhibitor cocktail [Sigma]). Lysates were precleared on protein G magnetic beads (New England Biolabs) followed by Myc-tagged protein complex capture on 9E10 monoclonal antibody-conjugated magnetic beads (MBL). The beads were washed with three times with lysis buffer, and bound proteins eluted in Laemmli buffer.

### *In vivo* mouse infection studies.

Groups of three C57BL/6J mice each weighing between 18 and 20 g were infected intraperitoneally with 1,000 tachyzoites of the RHΔ*ku80*, Δ*prp1*, or Δ*prp1* Δ*pgm2* strains. Following infection, mice were monitored daily for posture, activity level, and weight.

### Sequence analysis and phylogeny.

Phylogeny was performed using Geneious (80), and unrooted trees were plotted using the neighbor-joining algorithm.
