# Supplementary figures and images for: Two Phosphoglucomutase Paralogs Facilitate Ionophore-Triggered Secretion of the Toxoplasma Micronemes
Source: mSphere. 2017 Nov 29;2(6):e00521-17. doi: 10.1128/mSphere.00521-17 (PMC5705807; doi:10.1128/mSphere.00521-17)

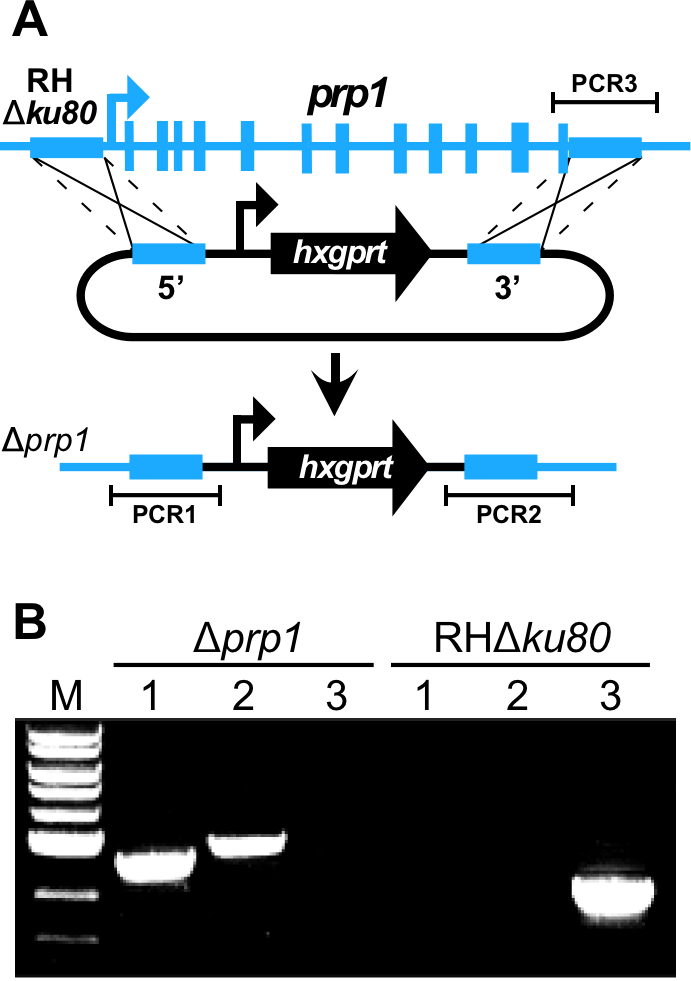

Supplement: FIG S1 [file sph006172414sf2.tif]

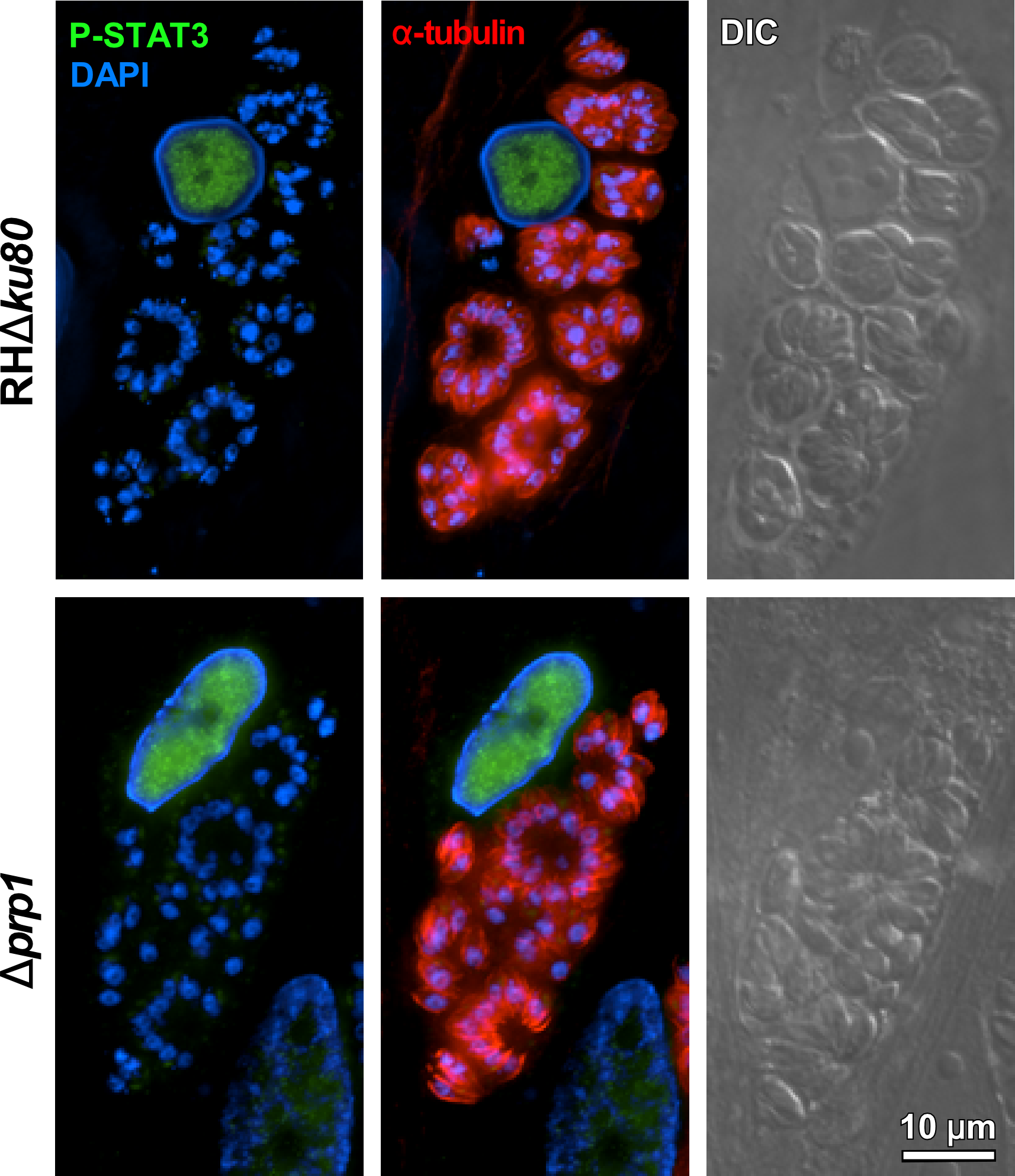

Supplement: FIG S2 [file sph006172414sf3.tif]

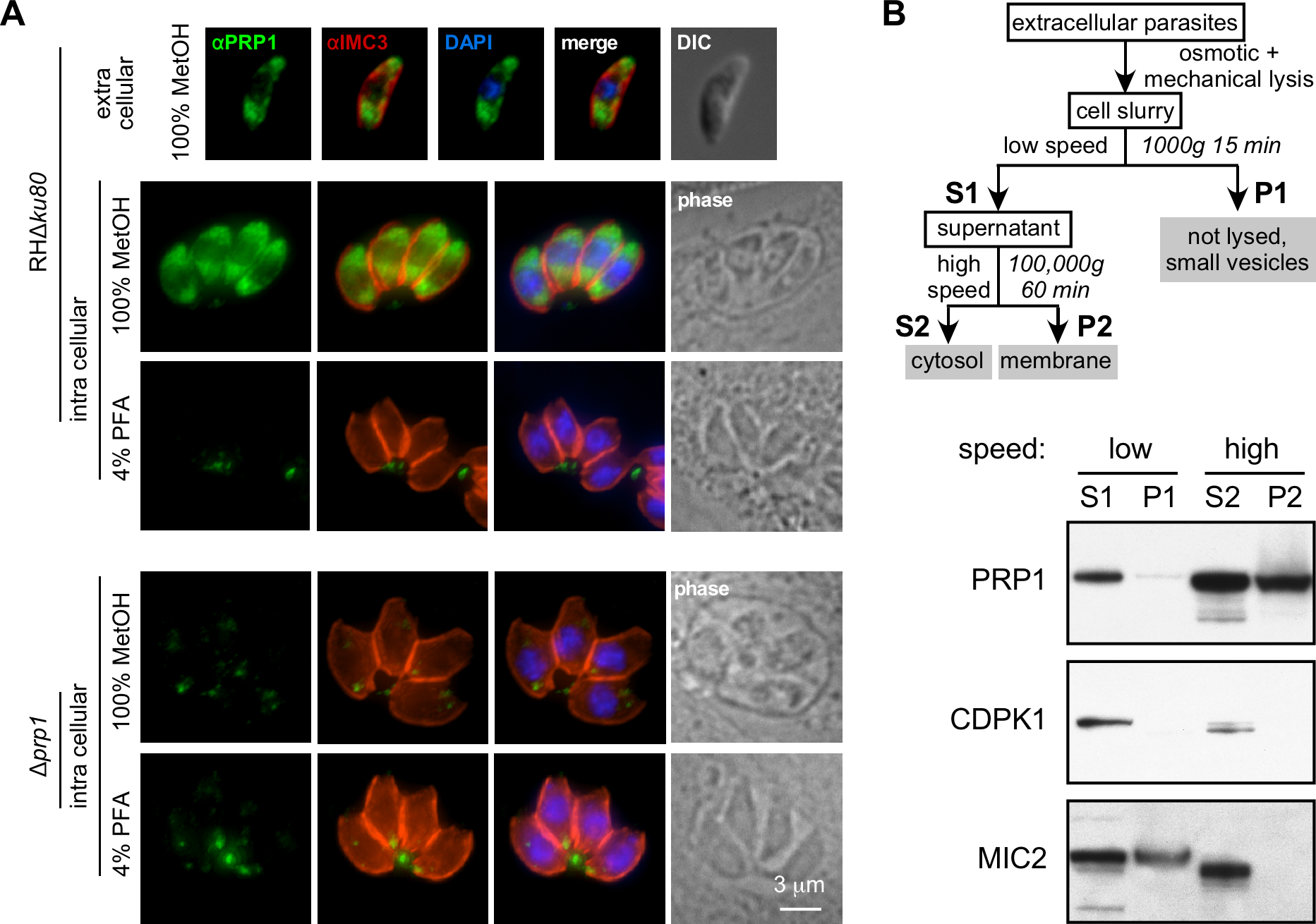

Supplement: FIG S3 [file sph006172414sf4.tif]

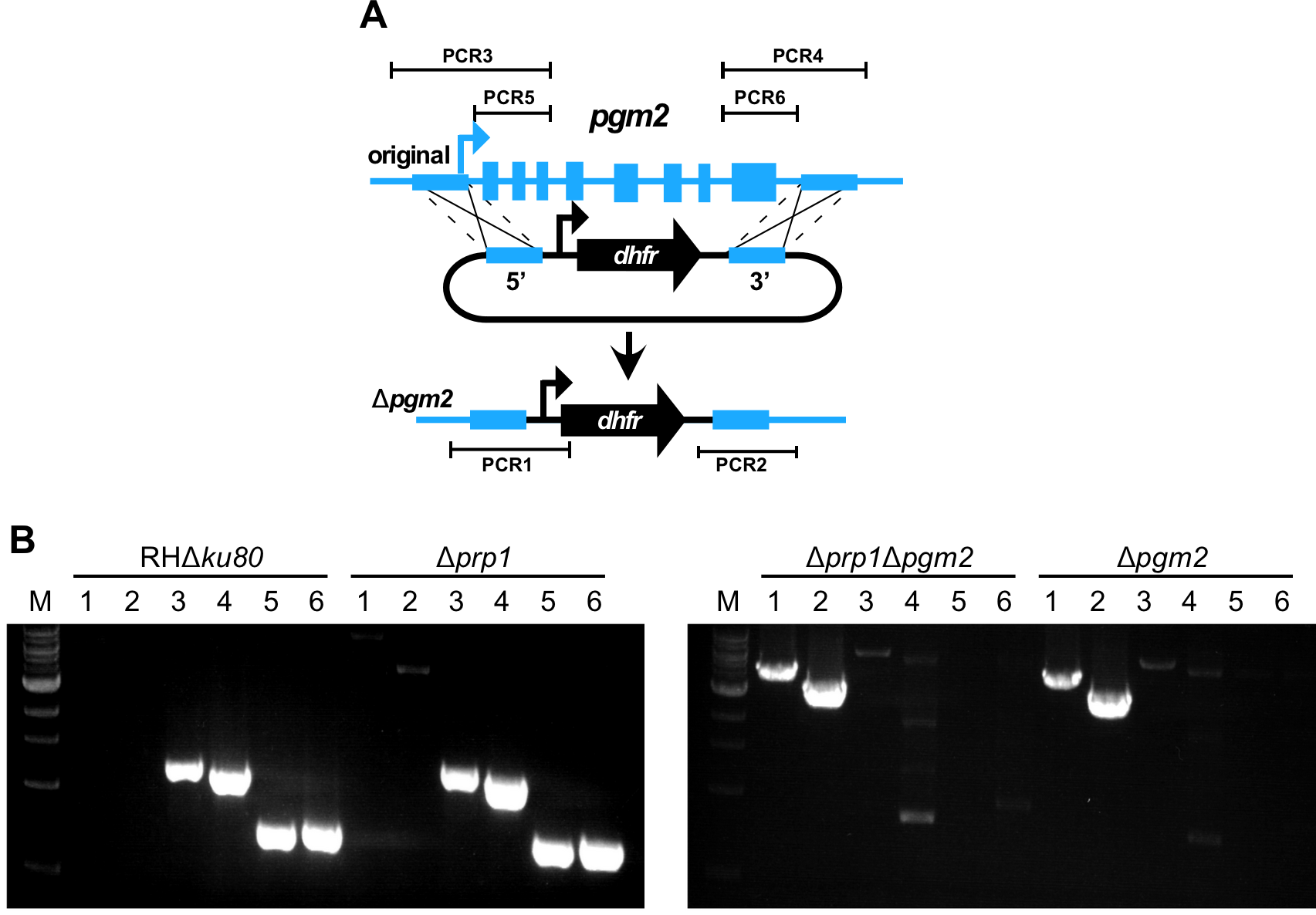

Supplement: FIG S4 [file sph006172414sf5.tif]

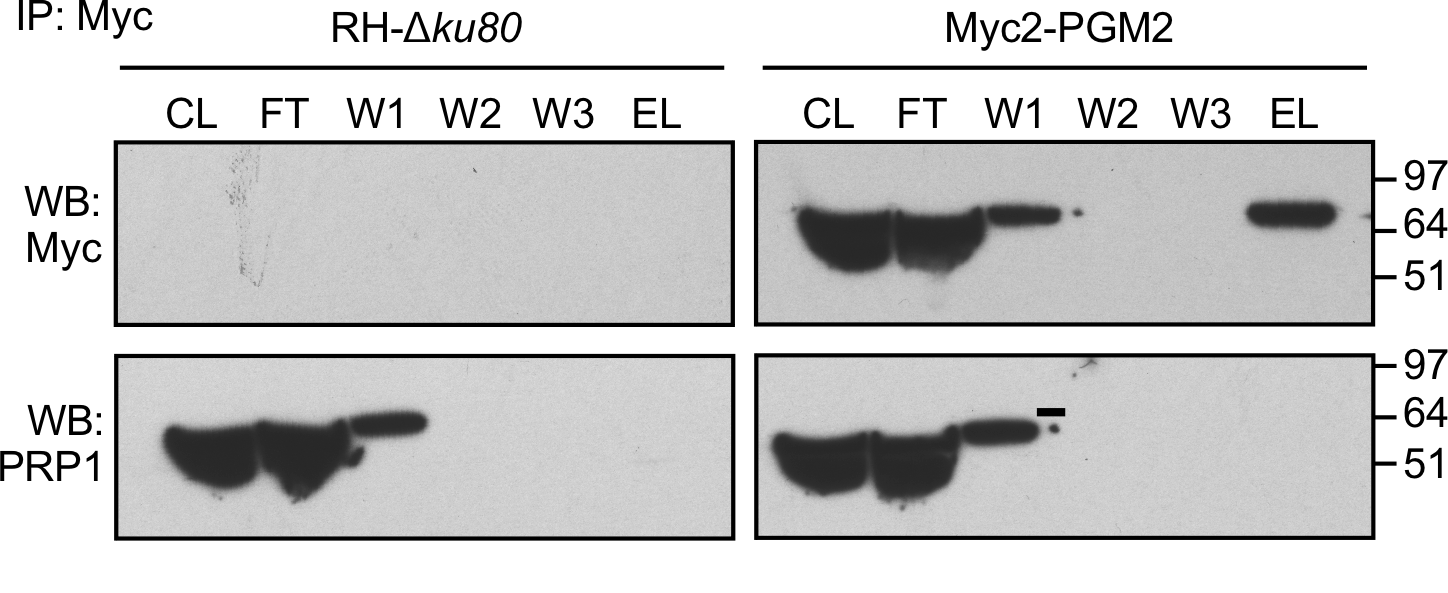

Supplement: FIG S5 [file sph006172414sf6.tif]
