# Supplementary material for: Two Phosphoglucomutase Paralogs Facilitate Ionophore-Triggered Secretion of the Toxoplasma Micronemes
Source: mSphere. 2017 Nov 29;2(6):e00521-17. doi: 10.1128/mSphere.00521-17 (PMC5705807; doi:10.1128/mSphere.00521-17)
Supplement: TABLE S1 [file sph006172414st1.pdf]

Table S1

Primer name	*Sequence (restriction enzyme sites underlined)*
Tagging of the PRP1 endogenous locus	
PRP1-LIC-F	TACTTCCAATCCAATTTAATGCCCAGCTACGAAGGAAACAGA
PRP1-LIC-R	TCCTCCACTTCCAATTTTAGCCGTAATGACAGTTGGCGTCT
PRP1-int-chk-F	CGAAGAGTCTTTCGGCACAGGAAGCG
Generation and verification of knockout of PRP1	
5'PRP1-KpnI-F	CAGGGTACCACGCAGAAAGTGCAGCTCTTG
5'PRP1-KpnI-R	CAGGGTACCGGAAGCCTCGGTTTCTTCC
3'PRP1-SacI-F	CAGGAGCTCGACGGGTGTTGCTGCTTTTTC
3'PRP1-SacI-R	CAGGAGCTCGAGAAACACTGTACTCGTACAAATG
Xho-tub-Fnsi	CAGCTC GAGGACATGCATGTCCCGCG
Xho-3dhfr-R	TCGCCTC GAGCTAGAACTAGCTAGTGGAC
PRP1-EcoRV-F	CAGGATATCAAAATGGGGGCCAAGGCAAGC
PRP1-EcoRV-R	CAGGATATCCGTAATGACAGTTGGCGTCT
HXGPRTseq-R	CCTTGCCCTTGCCGTAGTC
Generation of PRP1 specific antiserum	
aPRP1-LIC-F	GGGTCCTGGTTCGTGCATGGGTGATGTGGAG
aPRP1-LIC-R	CTTGTTCGTGCTGTTTATTACGTAATGACAGTTGGCGTC
Generation and verification of knockout of PGM2	
5'TgPGM2-UTR-F	GGCCGTTGTGTCCCACTTCCCGGTC ACTCGAGGTCGACGGTATCG
3'PGM2-UTR-R	GTGCACGCGTTAGATGCTTATGCGGCCGCTCTAGAACTAG
5PGM2-dKO-s	AAGTTGTCCGGTTCCCTCACAAGCGG
5PGM2-dKO-as	AAAACCGCTTGTGAGGGAACCGGACA
3PGM2-dKO-s	AAGTTGATCCAGGTCACACTCTGATG
3PGM2-dKO-as	AAAACATCAGAGTGTGACCTGGATCA
5'PGM2-dKO-int-F	GTCACGTAGACGGTGGACG
5'PGM2-dKO-int-R	GCTGAACTCACCTGGGTAG
3'PGM2-dKO-int-F	CATTTCCAGGAAAACGGCTGC
3'PGM2-dKO-int-R	CAACTCGACACCCGAAACAG
Generation of Myc2-tagged PGM2 expression plasmid	
PGM2-AvrII-F	CAGCCTAGGAAAATGTCTGTCGCAAACAGAAAG
PGM2-EcoRV-R	CAGGATATCTCAGAGTGTGACCTGGATCG
